# Digital Technologies for Symptom Monitoring in Parkinson Disease

**DOI:** 10.1007/s11910-026-01481-7

**Published:** 2026-03-04

**Authors:** Natalia Chunga, Varun Reddy, William Barbosa, Jamie L. Adams

**Affiliations:** 1 Department of Neurology, Louisiana State University Health Sciences Center at Shreveport, Shreveport, LA USA; 2 Center for Health + Technology, University of Rochester, Rochester, NY USA; 3 Department of Neurology, University of Rochester, Rochester, NY USA

**Keywords:** Parkinson disease, Digital technology, Wearable devices, Remote patient monitoring

## Abstract

**Purpose of Review:**

Digital health technologies (DHT) are promising tools for symptom monitoring in Parkinson disease (PD), offering objective and continuous data in real-life settings. This article reviews recent literature on DHT for symptom monitoring in PD, with a focus on remote monitoring devices.

**Recent Findings:**

Research studies have demonstrated that DHT can accurately and reliably monitor both motor and non-motor symptoms of PD, surpassing the limitations of subjective and episodic traditional clinical assessments. Digital measures show promise in predicting PD before clinical diagnosis, differentiating between individuals with and without PD, and detecting subtle symptom changes over time. Portable and non-invasive technologies—such as mobile applications, wearable sensors, and radio wave activity trackers—offer the opportunity to assess symptoms remotely, capturing day-to-day changes and real-world experiences.

**Summary:**

DHT have the potential to optimize monitoring of PD symptoms in clinical and research settings, which may help advance therapeutic development and tailor treatment interventions. As DHT continue evolving, standardization of the collection methods and selection of clinically relevant digital measures will be crucial for their wide-scale implementation.

## Introduction

With the growing prevalence of Parkinson disease (PD), efforts to develop disease-modifying therapies and tailored interventions have expanded. Nevertheless, PD diagnosis and symptom monitoring continue to be challenging, as they primarily rely on subjective scales and episodic clinician-based assessments. Standardized rating scales are commonly used, but they are limited by factors such as rater subjectivity and inter-rater reliability [1]. Moreover, previous studies have shown that PD pathology may begin years before the diagnosis, potentially reducing the efficacy of disease-modifying interventions due to late introduction in the disease course [2]. Additionally, clinician-based assessments only capture discrete, and typically short, periods of time, not accounting for day-to-day changes and real-world experiences of individuals with PD. These limitations underscore the need for better measures for early detection and symptom monitoring in PD.

In this context, digital health technologies (DHT) are promising tools for monitoring PD symptoms with objective, quantitative digital measures. The rapid expansion of portable, non-invasive, user-friendly devices has resulted in digital integration into our daily lives, enabling continuous monitoring in real-world settings. Increasing evidence shows the potential applications of DHT for monitoring PD symptoms, both for clinical and research purposes. In line with this, clinical trials in PD are already incorporating digital outcome measures with promising results [3, 4]. Nevertheless, the variability of technologies used, sampling methods, and study designs has limited the wide-scale implementation of DHT in PD. For this reason, there are ongoing efforts aimed at developing standardized recommendations to guide the selection of technologies and monitoring strategies, and to define clinically relevant digital measures [5].

In this review, we will discuss DHT for symptom monitoring in PD, with a particular focus on remote monitoring devices. We will first outline the technologies and methods used to collect digital measures, followed by a review of symptom-specific measures and recent studies in the field. While telehealth is considered a form of DHT, this topic will not be covered in this review.

## Digital Measures and Collection Methods

DHT are tools that leverage computing platforms, software, connectivity, and sensor technologies for healthcare and related uses [6]. Within this framework, digital measures are quantifiable physiological, behavioral, or clinical data that are collected through DHT. Digital measures can be designed to detect the presence of a disease (diagnostic), show response to a medical intervention (pharmacodynamic), or assess the status of a disease (monitoring) [7]. In PD, digital measures have excellent potential to provide longitudinal quantitative data to monitor motor and non-motor symptoms. In contrast to traditional clinical assessments, which are often infrequent and susceptible to subjectivity, digital measures allow for continuous assessment of daily symptoms and objective monitoring of disease progression and response to therapeutic interventions [8].

Digital measures can be collected using either active or passive monitoring strategies. Active monitoring requires that the individual engages in specific tasks (e.g., finger tapping data collected with a smartphone). In parallel, passive monitoring collects data automatically without participation or input of the individual (e.g., step count data collected by a wearable device). Various technologies can be used to collect these measures, and recent advances in DHT have allowed symptom monitoring to take place not only in the clinical setting but also at home [9]. In particular, there has been growing interest in using wearable sensors, smartphone applications, and radio wave activity tracers due to their ability to monitor symptoms remotely and in real-life environments [8]. Additionally, emerging artificial intelligence (AI) learning models enable the analysis of larger datasets, with the potential to provide greater sensitivity and specificity to change [10, 11]. In this section, we describe some of these technologies, highlighting a few recent notable studies and discussing limitations and challenges.

### Wearable Sensors

Wearable sensors (e.g., wrist-worn watches, smart rings, foot insoles, etc.) have become the most frequently used DHT for tracking PD progression in research studies, driving a rapid expansion of this field over the past decade [12]. Many wearable sensors have been developed, including inertial measurement units (IMU) and body-worn sensors (BWS). These technologies allow for continuous monitoring and provide comprehensive kinematic measures of complex motor behaviors using non-invasive methods [13].

In the study WATCH-PD (Wearable Assessment in The Clinic and at Home in PD), a smartwatch-smartphone system was used to examine individuals with early, untreated PD [14]. Over a 12-month period, decline in several gait and tremor digital measures was observed, with consistent findings on at-home versus in-clinic assessments, and good test-retest reliability. Remarkably, the change in digital measures often exceeded the corresponding change on individual items of the Movement Disorder Society Unified Parkinson’s Disease Rating Scale (MDS-UPDRS). Similarly, the Mobilise-D study used a single wearable sensor on the low back to measure and validate real-world gait metrics in various conditions affecting mobility, including PD [15]. Gait metrics from the wearable sensor were compared to a multi-sensor reference system and analyzed alongside in-clinic values. While laboratory performance remained superior, this study demonstrated that a single inexpensive wearable device could suitably provide gait metrics across a wide range of clinical conditions and environments. Additionally, the PDMonitor study used BWS at the wrist, torso, and ankles for continuous home monitoring of individuals with PD for up to seven days [16]. Device motor symptom detection was compared to MDS-UPDRS scores and participant event diaries, and demonstrated reliable accuracy for multiple metrics (including bradykinesia, gait, tremor, dyskinesia, and OFF periods).

### Mobile Applications

With the steady increase in smartphone users, there has been expanding interest in using mobile applications for healthcare purposes given portability, instantaneous access, and potential cost-effective solutions [17]. In PD, these applications provide an opportunity to track symptoms remotely with both active and passive monitoring methods.

The Roche-PD Mobile Application v2 is a noteworthy example [18]. This mobile application combines daily administered active tests with passive smartphone and smartwatch monitoring, providing measures of various PD symptoms (including bradykinesia, bradyphrenia, speech, tremor, gait, and balance). In a preliminary clinical validity study that included participants with early-stage PD, the Roche mobile application demonstrated good to excellent test-retest reliability over a two-week period (intraclass correlation coefficient [ICC] ≥ 0.75), and digital measures correlated with MDS-UPDRS scores.

### Radio Wave Activity Trackers

In efforts to improve the feasibility of at-home passive monitoring of symptoms and reduce the burden or potential complications that may arise from users being asked to manage devices, recent studies have utilized radio wave activity trackers.

This type of technology has been applied to monitor motor and non-motor PD symptoms. For instance, radio wave trackers were used to evaluate the at-home gait speed in participants with and without PD for up to 1 year [19]. Radio wave analysis showed a strong correlation with clinical assessment using the MDS-UPDRS and captured symptom fluctuations in response to medications. In parallel, another study showed that radio wave analysis of nocturnal breathing patterns, in combination with an AI model, was able to detect PD with good sensitivity and specificity [20]. This method also demonstrated improved disease progression tracking when compared to traditional clinical assessments. Both studies showed high test-rest reliability, with an ICC of 0.98 after 14 days and 0.95 after 12 nights of monitoring, respectively. As these wireless devices run continuously in the background of an individual’s home, they hold promise for monitoring PD symptoms during everyday activities and without change in typical behavior.

Radio wave activity trackers also face limitations and challenges. While they bring value for pattern recognition (e.g., gait, breathing), there is limited data for monitoring specific symptoms, including tremor and bradykinesia. Besides, monitoring is limited to the space covered by the radio device [19], which may be too confined to be truly representative of real-life daily activity and symptoms, particularly in early disease. Another consideration includes the potential impact of environmental and technical factors; for example, the presence of multiple individuals at home or use of electronic devices can affect the data collection, although newer algorithms have allowed to minimize these factors [19, 20].

## Motor Symptom Monitoring

PD is characterized by motor dysfunction, including cardinal signs of bradykinesia, tremor, rigidity, and gait and balance impairment, which remain essential for the clinical diagnosis of the disease. Nonetheless, the variability of PD motor symptoms with significant day-to-day fluctuations poses a challenge for the current episodic clinician-rated assessments [21]. Digital measures offer a potential solution by providing continuous remote monitoring of motor function, which can aid clinicians and researchers to better tailor treatments and develop validated and robust endpoints for clinical trials [22, 23]. The following section outlines symptom-specific digital measures for monitoring motor function in PD, and a selection of notable studies is summarized in Table 1.

**Table 1:** Selected digital health technology studies on Parkinson disease motor symptom monitoring

Symptom	Notable studies	Digital technology	Assessment	Digital measures	Selected results
Tremor	*Sigcha et al.*, [24]	Smartwatch-smartphone system, Monipar mobile health tool	Active Tasks: - Resting tremor - Postural tremor - Movement of hands to chest - Finger tapping - Rapid hand movements - Pronation-supination hand movements - Arising from a chair - Gait	Tremor, upper extremity bradykinesia, gait	Digital resting tremor data showed high correlation with clinician-based assessments (MDS-UPDRS).
	*Rodriguez et al.*, [25]	ZurichMove, machine learning model	Passive Monitoring	Tremor	Machine-learning displayed high accuracy in assessing tremor severity (tremor detection sensitivity was 0.9 and overall classification accuracy 0.88).
Bradykinesia	*Wu et al.*, [26]	Kinect-based motion analysis system, regression model	Active Tasks: - Finger tapping - Hand movements - Pronation-supination movements of hand - Toe tapping - Leg agility	Bradykinesia	Kinematic features showed significant correlations with clinical scales and exhibited significant decreases in multiple bradykinesia tasks. Regression model with combination of motor tasks showed high diagnostic value (AUC of 0.955).
	*Mishra et al.*, [27]	Kinesia One Sensor system	Active Tasks: - Hand grasp task - Arm pronation-supination	Upper extremity bradykinesia	Wearable sensors detected improvement of all subcomponents of upper extremity bradykinesia for people with PD with 180 Hz STN DBS and dopaminergic medication.
Gait and mobility	*Mirelman et al.*, [28]	Axivity, regression model	Passive Monitoring	Gait quality, nocturnal behavior, activity quantity and distribution patterns	Multiple mobility measures showed greater effect sizes than corresponding MDS-UPDRS score. Model with 14 digital measures able to accurately distinguish people with PD and controls (81.1%, AUC 0.87).
	*Sotirakis et al.*, [29]	OpalTM sensor, random forest model	Active Tasks: - Walking - Postural Sway	Gait-related features, posture	Random Forest Model detected statistically significant progression of motor symptoms within 15 months, while MDS-UPDRS part III did not capture change.
Hypomimia	*Novotny et al.*, [30]	Video-based analysis	Active tasks: - One minute of freely spoken monologue	Hypomimia markers of eight facial regions (forehead, nose root, eyebrows, eyes, lateral canthal areas, cheeks, mouth, jaw)	Hypomimia digital measures correlated with the total MDS-UPDRS part III score.
	*Adnan et al.*, [31]	Video-based analysis, machine learning model	Active tasks: - Mimicking disgust - Smile - Surprised expression	Facial action units, facial landmarks (eye and mouth openness, eyebrow raising, jaw drop, mouth width)	Facial features analysis distinguished between people with and without PD (accuracy: 87.9%, sensitivity: 76.8%, specificity: 1.4%).
Speech and voice	*Lipsmeier et al.*, [18]	Roche-PD mobile application	Active tasks: - Sustained phonation - Reading aloud	Jitter (phonation), monotonicity (prosody)	Monotonicity and jitter correlated with impairment of speech clinical scores in dopaminergic-treatment-naïve PD individuals.
	*Adams et al.*, [14]	Smartwatch-smartphone system, BrainBaseline™ mobile application	Active tasks: - Sustained phonation - Reading aloud	Pitch	Pitch reduction was noted in individuals with early PD before clinical changes in the MDS-UPDRS.
Motor fluctuations	*Fay-Karmon et al.*, [32]	Smartwatch-smartphone system, Intel ^**®**^ Pharma Analytics Platform	Passive monitoring. Active Tasks: - Static postural test - Static rest test - Pronation-supination - Finger tapping - Timed up and go	Tremor, dyskinesia, activity level, bradykinesia	Using smartwatch technology for home-based monitoring, motor-fluctuation profiles were characterized.

*AUC* area under curve, *DBS *deep brain stimulation, *MDS-UPDRS* Movement Disorder Society Unified Parkinson’s Disease Rating Scale, *PD* Parkinson disease, *STN* subthalamic nucleus

### Tremor

Tremor is considered one of the most prevalent and debilitating symptoms for people with PD (PwP) [33, 34]. Smartwatch-smartphone systems offer an emerging means to monitor tremor, allowing for both passive and active monitoring of resting and postural tremor, which can provide reliable quantification of disease severity [14, 18, 24]. Similarly, BWS have been proposed for tracking PD motor symptoms including tremor, with devices like PDMonitor^®^, Personal Kinetigraph^®^, and Kinesia One™ [35]. Among the BWS, a promising alternative includes the triboelectric sensors, which can generate its own electricity through mechanical motion enabling continuous, unobtrusive monitoring of tremor [36]. Commercially available devices can also capture tremor data; for example, the Apple watch detected significant changes in tremor measures in a cohort of individuals with early PD over the course of one year [14]. Furthermore, researchers have developed algorithms that have the ability to detect tremor and response to medications [37]. Using these technologies, digital measures of PD tremor can be obtained, including oscillations characteristics (e.g., frequency, amplitude), temporal burden metrics (e.g., percent of time with tremor), and medication response (e.g., ON-OFF differences).

As innovative sensing modalities are expanding, there has been a rising need for the development of methods to detect clinically relevant patterns from wearable sensor data in free-living environments. Machine learning algorithms offer a potential answer to this need with a recent model displaying a tremor detection sensitivity of 0.9 and classification accuracy of 0.88 for individuals with PD using wearable sensors [25]. A separate unsupervised model demonstrated an accuracy of 76% in classifying tremor versus non-tremor states [38].

### Bradykinesia

Bradykinesia is the hallmark feature of PD. However, there is considerable variability in clinician-based ratings [39]. Digital measures have the potential to objectively measure bradykinesia [18, 24, 35], typically with active monitoring of hand and leg movements (e.g., finger tapping, toe tapping), and fine motor tasks (e.g., handwriting, drawing a shape). A notable example is Monipar –a mobile health application for smartwatches-, which showed high correlations between digital measures and clinical assessments of motor symptoms, including bradykinesia and tremor [24]. Also, a study using the Roche-PD mobile application found that all three tests for bradykinesia (hand turning, draw a shape, dexterity) correlated with the corresponding MDS-UPDRS upper limb bradykinesia item scores [18]. In a similar way, BWS have been applied to assess bradykinesia and fine motor function; in the WATCH-PD study, the APDM Opal sensor was combined with a smartphone-smartwatch system, showing reduced longitudinal performance on fine motor tasks in PwP compared with controls [14]. Alongside their reliability, current devices have shown enhanced detection of treatment effects on bradykinesia [27, 40]. For instance, Kinesia sensor system demonstrated differential impacts on upper limb bradykinesia with high versus low levels of deep brain stimulation of the subthalamic nucleus in PwP [27].

Other methods for assessing bradykinesia include video-based technology like the Microsoft Kinect for Xbox One sensor, which captures 3D body motion data. This technology was able to detect differences in bradykinesia-related motor tasks in PwP compared to healthy controls [26]. Novel machine learning frameworks are also being developed to assess single-view and seconds-long videos (e.g., finger tapping) captured on consumer-grade devices—like tablets and smartphones—to classify motor severity in PD [41].

### Gait and Mobility

Wearable sensors, particularly IMU, enable real-time measurement of spatiotemporal gait patterns (e.g., cadence, velocity, stride length, stance) and provide detailed insights into pathological features, including freezing of gait [42]. IMU are able to act as suitable alternatives to the gold standard optical motion gait analysis systems [42]. Notably, digital measures collected by IMU demonstrate better detection and increased monotonicity in tracking the progression of gait and postural changes when compared to clinician-rated assessments [29].

Wearable triaxial accelerometers, such as the Axivity device, have also shown enhanced detection of prodromal and early-stage PD using real-world digital gait measures [28, 43, 44]. Similarly, the APDM Opal wearable sensor (which incorporates accelerometer and gyroscope data) detected changes in gait measures in a 12-month longitudinal study of early PD [14]. Using wearable accelerometers, a model based on 14 digital measures—including gait speed, number of steps, and velocity of rotation—showed an 81.1% accuracy in distinguishing between participants with and without PD [28]. Likewise, other validated algorithms developed from wearable sensor data have proven effective in tracking mobility performance (e.g., walking speed, daily walking duration) for individuals with gait impairments [15, 45]. Alongside providing a comprehensive characterization of gait function in daily life, the use of accelerometry data with machine learning models has shown better test performance in predicting PD up to 7 years before the diagnosis compared to other models based on genetics, lifestyle, blood biochemistry, or prodromal symptoms [44].

Concurrently, smartwatches have been tested for their reliability in detecting real-life ambulation in PD. Average daily steps collected by consumer smartwatches have been identified as a reliable measure for individuals with mild-to-moderate PD [46]. Also, for early PD, significant reductions in gait speed, arm swing, and stride length were noted in participants using a smartwatch over the course of one year [14]. Radio wave activity trackers are another effective approach for continuous monitoring of PD, providing passive data of at-home gait speed to assess progression [19].

### Hypomimia

Hypomimia is a highly prevalent PD sign, present in up to 92% of affected individuals, and it may develop years before the clinical diagnosis. As such, it is a promising marker for PD screening and symptom monitoring, and digital measures are a potential tool for automatic and objective measurement.

A study using an automated video-based analysis of facial features showed an accuracy of 78.3% at differentiating PwP from healthy controls, slightly higher than an expert assessment of hypomimia which had an accuracy of 75.9%. In addition, hypomimia digital measures showed significant correlations with the total MDS-UPDRS part III score, suggesting that hypomimia might be used as a surrogate of the overall severity of motor symptoms [30]. Along similar lines, a recent study combined video-based analysis of facial features with a machine learning model to distinguish between people with and without PD. This model achieved an accuracy of 87.9%, with a sensitivity of 76.8% and a specificity of 91.4%. The performance remained high when this method was tested on external datasets, with accuracies of 80.3% and 85.3% across two independent cohorts [31].

### Speech and Voice

Speech and voice changes are early symptoms of PD, holding value for detecting and monitoring disease severity over time. Digital measures may be obtained using different sampling methods—including spontaneous narrative discourse, reading passages, automatic speech, and standardized tasks [47]-, and smart devices and computer-based software enable accessible ways to collect data remotely.

A notable example is the Roche-PD mobile application, which includes remote monitoring of speech and voice through tasks of sustained phonation and reading aloud. In a reliability and validity study, digital measures of monotonicity and jitter correlated with impairment of speech clinical scores in dopaminergic-treatment-naïve PD individuals [18]. Similarly, in the multicenter study WATCH-PD, speech and voice were evaluated over a 12-month period using a smartwatch-smartphone system [14]. Digital measures derived from reading and phonation tasks differed between individuals with early, untreated PD and age-matched controls. Notably, pitch reduction was noted in PwP, even when MDS-UPDRS speech ratings were normal; nonetheless, progression was not detected over 1 year of monitoring [14]. In laboratory settings, machine learning models have been applied to analyze speech and voice in PD, with promising results that suggest potential for monitoring disease severity and response to medications [48, 49].

### Motor Fluctuations: OFF-ON States

Dopaminergic medications can mask PD progression signals and complicate assessments, which poses a significant challenge for clinical trials of disease-modifying therapies [50]. At the same time, motor fluctuations related to the medication states (OFF or ON) can impact daily function and quality of life. As noted before, DHT provide sensitive methods to detect symptoms changes, which may allow for accurate recognition of medication responses and fluctuations.

Smartwatches have been tested for home-based monitoring of individual motor fluctuations (tremor, dyskinesias, gait changes) and patient-specific features of OFF-ON states in PD, showing promise in their ability to act as pharmacodynamic response measures and improve individualized treatment [32, 40]. Other studies have combined gait data from wearable sensor devices was machine learning models, demonstrating that this method can accurately classify OFF and ON states in individuals with PD [51, 52]. Additionally, radio wave sensors have shown potential to identify medication responses based on analysis of gait patterns [19].

## Non-Motor Symptom Monitoring

Non-motor symptoms are highly prevalent in PD, often preceding the onset of motor manifestations. Although most studies on PD digital measures have primarily focused on motor symptoms, emerging evidence suggests their potential utility for non-motor symptoms as well, offering opportunities for remote assessments, early detection, and longitudinal evaluations in real-life settings. This is particularly important given that non-motor symptoms significantly drive quality of life in PwP [53]. The following section outlines symptom-specific digital measures for monitoring some of the non-motor features in PD, and a selection of notable studies is summarized in Table 2.

**Table 2:** Selected digital health technology studies on Parkinson disease non-motor symptom monitoring

Symptom	Notable studies	Digital technology	Assessment	Digital measures	Selected results
Sleep	*Yang et al.*, [20]	Radio wave tracker, machine learning model	Passive monitoring	Breathing patterns	This method showed a sensitivity of 86.23% and specificity of 82.83% to detect PD. Longitudinal analysis also demonstrated improved disease progression tracking compared to clinician assessments.
	*Raschellà et al.*, [54]	GENEActiv Original^®^ wrist actigraph, machine learning model	Passive monitoring	Movement episodes, global movement patterns	RBDAct screening tool was developed using the machine learning model. Over a 2-week period of at-home monitoring, this tool showed an accuracy of 100% for diagnosing RBD in individuals with PD and 94% in controls.
Cognitive symptoms	*Lipsmeier et al.*, [18]	Roche-PD mobile application	Active task: - Electronic Symbol Digit Modalities Test	Psychomotor slowing	Good test-retest reliability was shown for the electronic version of the Symbol Digit Modalities Test, but correlation with the MDS-UPDRS cognitive impairment score was weak.
	*Adams et al.*, [14]	Smartwatch-smartphone system, BrainBaseline™ mobile application	Active task: - Digital Trail Making Test	Psychomotor slowing	Reduced scores were noted at baseline in people with PD compared to healthy controls, but no changes in the digital measures were noted over time.
	*Ferrante et al.*, [55]	Web-based TELL app, machine learning model	Active tasks: - Taxonomic: mention names of animals - Thematic: mention supermarket items - Phonemic: produce words that begin with /p/	Digital word features (semantic variability, granularity, concreteness, length, neighbors, frequency)	Digital word property analysis predicted dementia scores in people with PD and differentiated between those with and without mild cognitive impairment. Semantic variability, granularity, frequency, and concre150415teness were the main distinctive features.

*MDS-UPDRS* Movement Disorder Society Unified Parkinson’s Disease Rating Scale, *PD* Parkinson disease, *TELL* toolkit to Examine Lifelike Language, *RBD* REM-sleep behavior disorder

### Sleep

Sleep disturbances are common in PD, including REM-sleep behavior disorder (RBD) which often precedes the diagnosis by several years. Passive monitoring using wearable sensors can provide valuable information about sleep patterns. For example, the GENEActiv Original^®^ wrist actigraph was used to collect data on nocturnal movements, which was then combined with a machine learning model to develop the RBDAct screening tool. RBDAct had an accuracy of 92% for diagnosing RBD in the clinic; with at-home assessments over a 2-week period, accuracy was 100% in individuals with PD and 94% in controls [54].

Digital measures of sleep have also shown potential for early detection of PD. Yang et al. used an AI model derived from a large dataset of 7,671 individuals across multiple sites to analyze the radio wave breathing patterns during sleep of 99 participants with and without PD. Using this model, PD was detected with a sensitivity of 86.23% and specificity of 82.83%. Longitudinal analysis further demonstrated that this approach outperformed clinician assessments in tracking disease progression over six- and twelve-month intervals [20].

### Cognitive Symptoms

Nearly half of individuals with PD experience cognitive symptoms at any given time, and up to 80% develop dementia in the advanced stages [56]. Digital measures could help improve early detection and longitudinal monitoring of cognition in daily living environments. Well-known cognitive assessments have been incorporated into mobile devices and applications. In the WATCH-PD study, digital versions of the Trail Making Test were included. At baseline, reduced scores were noted in PwP, although no progression was captured over time [14]. In a similar way, the Roche-PD mobile application used the electronic Symbol Digit Modalities Test to measure psychomotor slowing. This tool showed a good test-retest reliability but only associated weakly with the MDS-UPDRS cognitive impairment Section [18].

Additionally, digital measures of language and movement patterns might help monitor cognitive symptoms. In a recent study, digital word property analysis was used to predict cognitive performance in PwP. Spoken language samples obtained through verbal fluency tasks were analyzed and subsequently used as inputs for a machine learning model. This model predicted cognitive scores in PwP and differentiated between those with and without mild cognitive impairment [55]. Furthermore, a longitudinal study applied non-invasive home sensors to passively monitor movement complexity over a 10-year period. Digital measures combined with machine learning algorithms were found to predict cognitive changes in older adults [57]. While not specific to PD, the findings suggest that passive monitoring of movement complexity holds potential for detecting cognitive decline, and it could be applied in future PD research.

### Psychiatric Symptoms

As psychiatric symptoms are common in PD, anxiety and depression rating scales are frequently used for clinical and research purposes. Their brief and self-report formats may be suitable for electronic or remote administration, although no validation studies of the digital versions were found in our literature review.

Digital physiological measures have been studied for detecting anxiety and depression. For instance, data collected with a Fitbit Charge 2^®^ wrist-worn sensor showed that greater variation of nighttime heart rate and lower regularity of weekday circadian rhythm were associated with depressive symptoms in healthy adults [58]. Ambient speech analysis with smartphone applications has also been studied for the evaluation of psychiatric symptoms. Using this technology, a previous study showed that digital measures of language properties were associated with depression and anxiety [59]. These tools have not been previously studied in PD, but future studies could consider their application.

## Current Challenges and Future Directions

While the technologies described offer many advantages and opportunities in PD, there remain significant challenges. Depending on the monitoring method approach, specific strengths and limitations for data collection may arise. Active monitoring methods allow for symptom-specific measures that can be directly compared with traditional clinical assessments, and data collection tends to be more structured. However, they typically capture discrete data points, limiting the evaluation of symptom fluctuations, and require user engagement which may increase participant burden. In contrast, passive monitoring methods enable continuous data collection, capturing symptom fluctuations and data that is more consistent with everyday experience, and typically have less user burden. Nonetheless, data consistency may be compromised by factors such as device non-use and operating system updates [60]. Passive monitoring in the free-living environment, for example with radio wave activity tackers, also raises concerns related to privacy and data security for both participants and those in close proximity, potentially impacting the overall acceptability of these technologies [61, 62]. In addition, maintaining long-term participant consent for passive monitoring technologies can be difficult [60].

Participant adherence represents a challenge for both active and passive monitoring methods. For instance, adherence to symptom reporting on a mobile application was 44% in average over a 6-month period, with less than 40% at 5–6 months [63]. Similarly, adherence to wearable sensors may reduce with longer periods of monitoring, especially for those with no previous experience using this type of devices [64]. Still, drops in adherence can be overcome by providing accessible study team support for technology issues, actively engaging participants, and providing results back as able. This was successfully shown in the WATCH-PD study, where the adherence remained high at 70% over a 12-month period [14]. Additional barriers to DHT include technology issues, such as poor wireless connectivity and limited device storage capacity [62]. Moreover, DHT can generate large, complex datasets that are often contaminated by irrelevant factors (e.g., idiosyncratic behaviors, comorbid conditions, environmental factors), making data management challenging and requiring hybrid analysis approaches and multidisciplinary teams for appropriate data interpretation [62, 64]. Combining active and passive monitoring methods, implementing targeted strategies, and using machine learning models may help optimize the data collection and overcome these challenges in the future [60].

Additionally, the methodology heterogeneity of DHT studies remains an issue, including variability in device selection, data collection protocols, sampling models, and digital measures [8]. This makes it challenging for the data to be comparable and, hence, to be implemented on a wide scale. Given the rapid pace at which new digital tools are emerging, standardization of digital measures—rather than devices—has been proposed as a more sustainable strategy [65]. At the same time, with larger amounts of data being captured by new technologies, the standardization of clinically meaningful and patient-centered measures is a priority [66, 67]. Importantly, the FDA has emphasized the need for “fit-for-purpose” validation, in which DHT (both hardware and software) are evaluated for technical performance, reliability, and clinical relevance in the context of their intended use [6, 65]. Appropriate validation of DHT will also allow for the derived measures to be comparable across different devices. Given these complexities, coordinated collaboration among diverse stakeholders—including tech, pharmaceutical, healthcare, academic, and patient organizations—could facilitate the successful deployment and wide-scale implementation of DHT [65]. This would be particularly helpful to align DHT with PD clinical trial efforts, especially since digital measures may allow for more sensitive methods for tracking disease progression. This, in turn, could improve the statistical power of clinical trials and help move forward the development of therapeutic interventions.

Alignment with FDA regulatory requirements may also facilitate broader reimbursement pathways and health insurance coverage, resulting in more equitable access to DHT. However, it is important to note that limited digital literacy and unreliable access to technology remain barriers for DHT implementation, which could potentially accentuate already existing healthcare disparities if not addressed [62, 68]. These are important aspects to consider given potential benefits of integrating DHT into routine PD care. Digital measures could provide valuable insights into day-to-day symptom fluctuations, and technologies could enable real-time data sharing between patients and clinicians, potentially resulting in earlier interventions for changing or new symptoms. Continuous monitoring of progression might also prove helpful to provide anticipatory guidance in an individualized way; for example, detection of gait changes could inform early physical therapy interventions in those at risk of falls. With further validation and standardization, DHT could be integrated into routine care to support personalized treatment and improve quality of life.

Finally, as DHT continue to evolve, ongoing evaluation of privacy and security concerns will remain critical. It is also important to note that, while many digital tools are portable and non-invasive, interaction with these technologies may result in behavioral changes at the individual and group level. As newer forms of monitoring and devices are introduced, it will be essential to understand the user experience, including the acceptability and adherence with these monitoring methods.

## Conclusions

As highlighted in this review, DHT and digital measures have the potential to transform PD detection and symptom monitoring. By offering objective data that can be collected remotely, continuously, and in real-world settings, they surpass the limitations of traditional clinical assessments. The implementation of DHT in clinical trials could enable earlier detection and better monitoring of PD progression with cost-effective, non-invasive methods. By improving participant screening, recruitment, and outcome measures, DHT may help accelerate therapeutic development. In clinical practice, digital tools could improve data sharing, allow for timely interventions, and enhance personalized treatments to improve quality of life of PwP.

Wide scale implementation of DHT in PD will require coordinated efforts to address the challenges and limitations previously outlined. In addition, while research on these technologies has expanded substantially in the past decade, data on digital measures for non-motor symptoms remains limited. Future studies are needed to address this gap and be able to provide more comprehensive assessments of PD. Despite these limitations, the ever-evolving digital technologies hold enormous potential to optimize symptom monitoring both in the clinical and research settings, helping advance therapeutic development and improve care for individuals with PD.

## Key Reference

- Espay AJ, Hausdorff JM, Sanchez-Ferro Á, Klucken J, Merola A, Bonato P, et al. A Roadmap for Implementation of Patient-Centered Digital Outcome Measures in Parkinson’s disease Obtained Using Mobile Health Technologies. Mov Disord Off J Mov Disord Soc. 2019;34:657–63.
  - Position paper from the Movement Disorders Society Task Force on Technology that outlines a framework to develop patient-centered digital measures both for clinical and research purposes.
- Mirelman A, Siderowf A, Chahine L. Outcome Assessment in Parkinson Disease Prevention Trials: Utility of Clinical and Digital Measures. Neurology. 2022;99:52–60.
  - Review paper that describes the approaches to measure PD motor performance in the prodromal phase, highlighting the ability of digital measures to detect subtle motor features and potential value in prevention clinical trials.
- Lipsmeier F, Taylor KI, Postuma RB, Volkova-Volkmar E, Kilchenmann T, Mollenhauer B, et al. Reliability and validity of the Roche PD Mobile Application for remote monitoring of early Parkinson’s disease. Sci Rep. 2022;12:12081.
  - Observational study that assessed the Roche PD mobile application v2 for remote monitoring of symptoms in dopaminergic-treatment- naïve PD individuals, showing preliminary evidence that supports the reliability and clinical validity of at-home motor digital measures.
- Adams JL, Kangarloo T, Gong Y, Khachadourian V, Tracey B, Volfson D, et al. Using a smartwatch and smartphone to assess early Parkinson’s disease in the WATCH-PD study over 12 months. NPJ Park Dis. 2024;10:112.
  - Longitudinal multicenter observational study that evaluated digital measures in early PD, showing significant change over time, most notably in gait and tremor digital measures.
- Mammen JR, Lerner A, Al-Rubayie R, Kostrzebski M, Stephenson D, Xiao Y, et al. Longitudinal qualitative assessment of meaningful symptoms and relevance of WATCH-PD digital measures for people with early Parkinson’s. J Neurol. 2025;272:114.
  - Longitudinal qualitative study that aimed to evaluate personally meaningful symptoms and relevance of digital measures to monitor what matters to people with PD, which was conducted alongside the ongoing multicenter WATCH-PD2 study. Results suggested that digital measures are relevant to people with PD, although some important aspects are not being captured and additional measures are needed.

## Data Availability

No datasets were generated or analysed during the current study.
